# Immune Evasion by *Yersinia enterocolitica*: Differential Targeting of Dendritic Cell Subpopulations *In Vivo*

**DOI:** 10.1371/journal.ppat.1001212

**Published:** 2010-11-24

**Authors:** Stella E. Autenrieth, Tanja-Rebecca Linzer, Clara Hiller, Birgit Keller, Philipp Warnke, Martin Köberle, Erwin Bohn, Tilo Biedermann, Hans-Jörg Bühring, Günter J. Hämmerling, Hans-Georg Rammensee, Ingo B. Autenrieth

**Affiliations:** 1 Interfakultäres Institut für Zellbiologie, Universität Tübingen, Tübingen, Germany; 2 Institut für Medizinische Mikrobiologie und Hygiene, Universität Tübingen, Tübingen, Germany; 3 Universitäts-Hautklinik, Universität Tübingen, Tübingen, Germany; 4 Medizinsche Universitätsklinik, Abteilung Innere Medizin II, Universität Tübingen, Tübingen, Germany; 5 Abteilung Molekulare Immunologie, Deutsches Krebsforschungszentrum (DKFZ), Heidelberg, Germany; 6 Interfakultäres Institut für Zellbiologie, Abteilung Immunologie, Universität Tübingen, Tübingen, Germany; University of Washington, United States of America

## Abstract

CD4^+^ T cells are essential for the control of *Yersinia enterocolitica* (Ye) infection in mice. Ye can inhibit dendritic cell (DC) antigen uptake and degradation, maturation and subsequently T-cell activation *in vitro*. Here we investigated the effects of Ye infection on splenic DCs and T-cell proliferation in an experimental mouse infection model. We found that OVA-specific CD4^+^ T cells had a reduced potential to proliferate when stimulated with OVA after infection with Ye compared to control mice. Additionally, proliferation of OVA-specific CD4^+^ T cells was markedly reduced when cultured with splenic CD8α^+^ DCs from Ye infected mice in the presence of OVA. In contrast, T-cell proliferation was not impaired in cultures with CD4^+^ or CD4^−^CD8α^−^ DCs isolated from Ye infected mice. However, OVA uptake and degradation as well as cytokine production were impaired in CD8α^+^ DCs, but not in CD4^+^ and CD4^−^CD8α^−^ DCs after Ye infection. Pathogenicity factors (Yops) from Ye were most frequently injected into CD8α^+^ DCs, resulting in less MHC class II and CD86 expression than on non-injected CD8α^+^ DCs. Three days post infection with Ye the number of splenic CD8α^+^ and CD4^+^ DCs was reduced by 50% and 90%, respectively. The decreased number of DC subsets, which was dependent on TLR4 and TRIF signaling, was the result of a faster proliferation and suppressed de novo DC generation. Together, we show that Ye infection negatively regulates the stimulatory capacity of some but not all splenic DC subpopulations in vivo. This leads to differential antigen uptake and degradation, cytokine production, cell loss, and cell death rates in various DC subpopulations. The data suggest that these effects might be caused directly by injection of Yops into DCs and indirectly by affecting the homeostasis of CD4^+^ and CD8α^+^ DCs. These events may contribute to reduced T-cell proliferation and immune evasion of Ye.

## Author Summary

Dendritic cells (DCs) are crucial in promoting immune responses against pathogens. Mouse DCs consist of different subpopulations but their role in immunity to pathogens and immune evasion is largely unclear. The enteric pathogen *Yersinia enterocolitica* (Ye) was shown to evade DC functions in bone marrow-derived DCs *in vitro* inhibiting antigen uptake and degradation, maturation and subsequently T-cell activation. However, it is controversial whether and, if so, which virulence factors of Ye (e.g. Yops) contribute to immune evasion of DCs *in vivo*. Using an experimental mouse infection model and a β-lactamase reporter system to track Yop injection into host cells we demonstrate here for the first time that distinct DC subpopulations are affected by Ye infection *in vivo* in terms of antigen uptake and degradation, cytokine production, and T-cell proliferation. Moreover, Ye infection causes the loss of 90% of CD11c^hi^CD4^+^ DCs in a TLR4- and TRIF-signaling dependent manner. These data combined with results reported from infection with e.g. *Mycobacterium tuberculosis*, *Salmonella typhimurium*, or *Escherichia coli* suggest that the response of DCs to bacterial infections is manifold, reflecting the diversity of DC subpopulations, pathogenicity factors, and life styles of the pathogens.

## Introduction

Host defense against microbial pathogens relies on the concerted action of both antigen-independent innate immunity and antigen-specific adaptive immunity [1]–[3]. Key features of the innate immune system include the ability to rapidly recognize pathogens and/or tissue injury and to signal the presence of danger to cells of the adaptive immune system [4]. Innate immune cells use a variety of receptors to recognize patterns shared between pathogens, e.g. bacterial LPS [5]–[7].

Dendritic cells (DCs) are unique antigen presenting cells that are able to induce primary immune responses, thus permitting the establishment of immunological memory [8]–[11]. Immature DCs are specialized to endocytose antigens [11]. Engagement of toll-like receptors (TLRs) expressed by DCs induces maturation and migration of DCs to secondary lymphoid organs where the antigens are presented to T cells in order to initiate adaptive immune responses. DC maturation is associated with reduced antigen uptake, up-regulation of MHC class II and costimulatory molecules and increased ability to prime T cells [12], [13]. Mouse splenic conventional CD11c^hi^ DCs can be subdivided according to surface marker expression into CD4^+^, CD8α^+^, and CD4^−^CD8α^−^ DCs [14]. The administration of LPS in mice causes migration of CD4^+^, CD8α^+^, and CD4^−^CD8α^−^DCs from the marginal zone into the T-cell zones of the spleen and is associated with apoptosis of DCs [15]–[17]. All splenic DC subpopulations can prime naïve T cells. CD8α^+^ DCs induce predominantly Th1 responses, while CD8α^−^ DCs promote Th2 responses [18]. CD8α^+^ DCs seem to be specialized for priming cytotoxic CD8^+^ T cells [19], [20].

*Yersinia enterocolitica* (Ye) is a Gram-negative predominantly extracellularly located bacterium that causes food borne acute or chronic gastrointestinal and systemic diseases [21]. Ye invades through M cells of the Peyer's Patches and may eventually disseminate to the lymph nodes, spleen, lung, and liver. The pathogenicity of Ye depends on the type three secretion system (TTSS) by which virulence factors, the so-called *Yersinia* outer proteins (Yops) are injected into the cytosol of host cells [22]. YopE, YopT and YopO modulate the cytoskeleton of the host cells, and YopH dephosphorylates focal adhesion molecules thereby inhibiting phagocytosis [23]–[26]. YopP inhibits NF-κB and MAP kinase signaling pathways and induces apoptosis in macrophages and bone marrow-derived DCs (BM-DCs) *in vitro* [27]–[33].

Based on an experimental Ye mouse infection model it was demonstrated that CD4^+^ and CD8^+^ T cells are required for control of Ye infection. Accordingly, T-cell deficient mice do not control Ye and die from a fulminant infection. Moreover, the adoptive transfer of Ye-specific CD4^+^ or CD8^+^ T cells into T-cell deficient mice confers protection against Ye infection [34], [35]. IFN-γ, TNF, IL-12 and IL-18 are essential for the control of Ye infection [36]–[39], suggesting a critical role of Th1 responses that may activate macrophages via IFN-γ production.

In previous *in vitro* studies we could show that Ye modulates specific immune functions of BM-DCs including their maturation [27], antigen uptake [40], processing [41] and subsequently CD4^+^ T-cell activation [27] by Yop injection. YopP induces apoptosis via the activation of caspases, and triggers caspase-independent necrosis in BM-DCs [27], [33]. Moreover, Ye YopP inhibits the up-regulation of costimulatory molecules and the production of proinflammatory cytokines [27]. It was also shown that YopP impairs clathrin-mediated endocytosis by BM-DCs and antigen degradation [40], [41]. Thus, Ye, particularly YopP, inhibits many functions of BM-DCs *in vitro*. However, Ye infection is usually a self-limiting disease and the question arises whether such profound inhibition of DC functions also occurs upon Ye infection *in vivo*. Furthermore, disruption of YopP in Ye does not significantly affect virulence of Ye *in vivo* thereby challenging the physiological relevance of the observations obtained by BM-DCs based *in vitro* studies [42]. In this study we analyzed the effect of Ye infection on splenic DC subpopulations. Our data show a functionally impaired CD8α^+^ DC subset regarding antigen uptake and processing associated with a reduced ability to activate CD4^+^ T cells. Moreover, Ye infection directly affected a major fraction of splenic DCs by injection of Yops leading to reduced expression of MHC class II and CD86 on CD8α^+^ DCs. Finally Ye infection caused a TLR4-TRIF-dependent indirect loss of CD4^+^ and CD8α^+^ DCs, which resulted from faster proliferation and inhibited *de novo* generation of these DC subpopulations.

## Results

### Ye reduces CD4^+^ T cell proliferation *in vivo*

**Figure 1:**
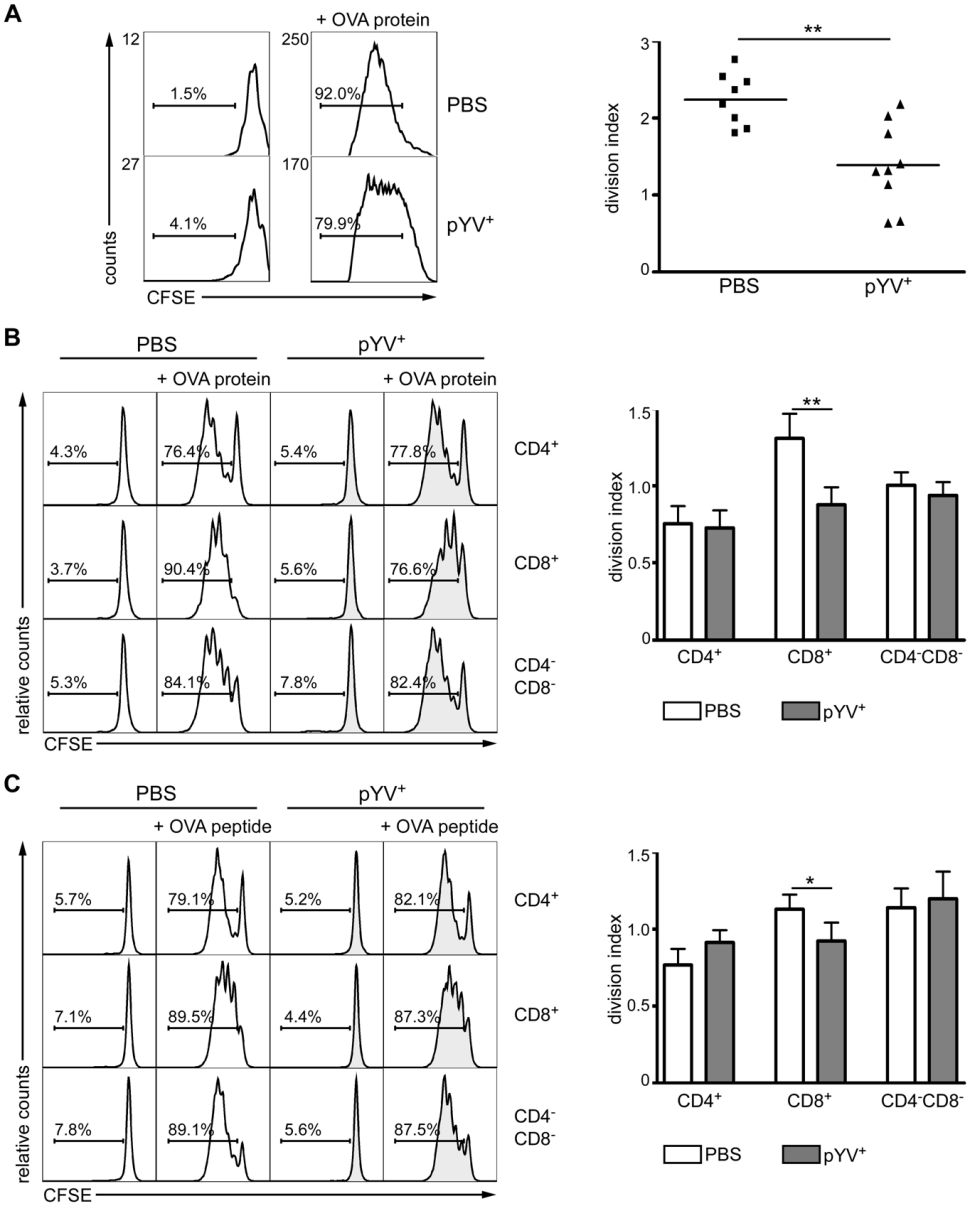
Infection of mice with Ye reduces CD4 T cell proliferation. (**A**) C57BL/6 mice were injected i.v. with 5×10^4^ Ye pYV^+^ or PBS. 6 h later 2×10^6^ CFSE-labeled CD4^+^ T cells from OT-II mice were adoptively transferred. 24 h p.i. OVA protein was administered i.v. and T-cell proliferation of adoptively transferred T cells in the spleen was analyzed 72 h later by flow cytometry. Histograms show CFSE dilution of 7-AAD^−^CD4^+^Vα2^+^ cells representative of three experiments with three mice per group. The percentage of cells below the marker is indicated. The diagram shows the division index (average number of cell divisions that the responding cells underwent (excluding undivided cells)) of the CD4^+^ T cells from PBS treated and Ye infected mice. The data shown are the summary of three independent experiments with three mice per group. (**B & C**) C57BL/6 mice were infected as described in (A). 24 h p.i. CD4^+^, CD8α^+^, and CD4^−^CD8α^−^DCs were sorted, and co-cultured *in vitro* with CFSE-labeled CD4^+^ T cells from OT-II mice without or in the presence of either OVA protein (**B**) or OVA peptide (**C**) for 72 h. The percentages of cells below the marker are depicted in the histograms. The diagrams show the division index of CD4^+^ T cells as described in (A). Data show quadruplicates from one out of two independent experiments. * indicates statistically significant differences.

T-cell responses are essential for the elimination of Ye [34], [35]. Accordingly, an increasing body of evidence derived from *in vitro* studies indicates that *Yersinia* may evade immune response by targeting DCs which are essential for triggering T-cell responses. To address whether Ye infection alters the capacity of DCs to induce T-cell proliferation *in vivo*, mice were infected with Ye wild type strain pYV^+^ followed by adoptive transfer of CFSE-labeled CD4^+^ T cells from anti-ovalbumin (OVA) T-cell receptor transgenic mice [43]. 24 h post infection (p.i.) OVA protein was injected intravenously (i.v.) into the mice. CFSE-dilution of proliferating CD4^+^ T cells was analyzed by flow cytometry three days later. OVA-specific CFSE-labeled CD4^+^ T cells showed no signs of proliferation in the absence of OVA after *in vivo* transfer. Infection of mice with Ye significantly reduced the number of proliferating antigen-specific T cells *in vivo* compared to that of PBS-injected control mice (Fig. 1A). Quantitative analysis of CFSE-dilution revealed a significant reduction of the division of OVA-specific CFSE-labeled CD4^+^ T cells from Ye-infected mice compared to PBS-treated mice (Fig. 1A). These data suggested that Ye affects the ability of DCs to activate CD4^+^ T cells *in vivo*.

To test whether a deficient DC maturation is responsible for the impaired T-cell proliferation, splenic CD4^+^, CD8α^+^, and CD4^−^CD8α^−^ DC subpopulations were sorted 24 h p.i. with Ye and co-cultured with OVA-specific CFSE-labeled CD4^+^ T cells in the presence of OVA protein (Fig. 1B) or OVA peptide (Fig. 1C). CD8α^+^ DCs from PBS-treated mice showed more pronounced CD4^+^ T-cell proliferation than CD4^+^ and CD4^−^CD8α^−^ DCs when co-cultured with OVA protein. Interestingly, the *Yersinia* infection significantly reduced the proliferation of the OVA-specific CD4^+^ T cells co-cultured with OVA protein (p = 0.0047) or peptide-pulsed (p = 0.037) CD8α^+^ DCs. In contrast, the T-cell proliferation capacity of CD4^+^ and CD4^−^CD8α^−^ DCs from Ye infected mice was unaffected when compared to controls (Fig. 1B & C). These data suggest that Ye affects antigen uptake and/or processing by and maturation of CD8α^+^ but not CD4^+^ and CD4^−^CD8α^−^ DCs.

### Ye modulates antigen uptake and degradation by DC subpopulations

**Figure 2:**
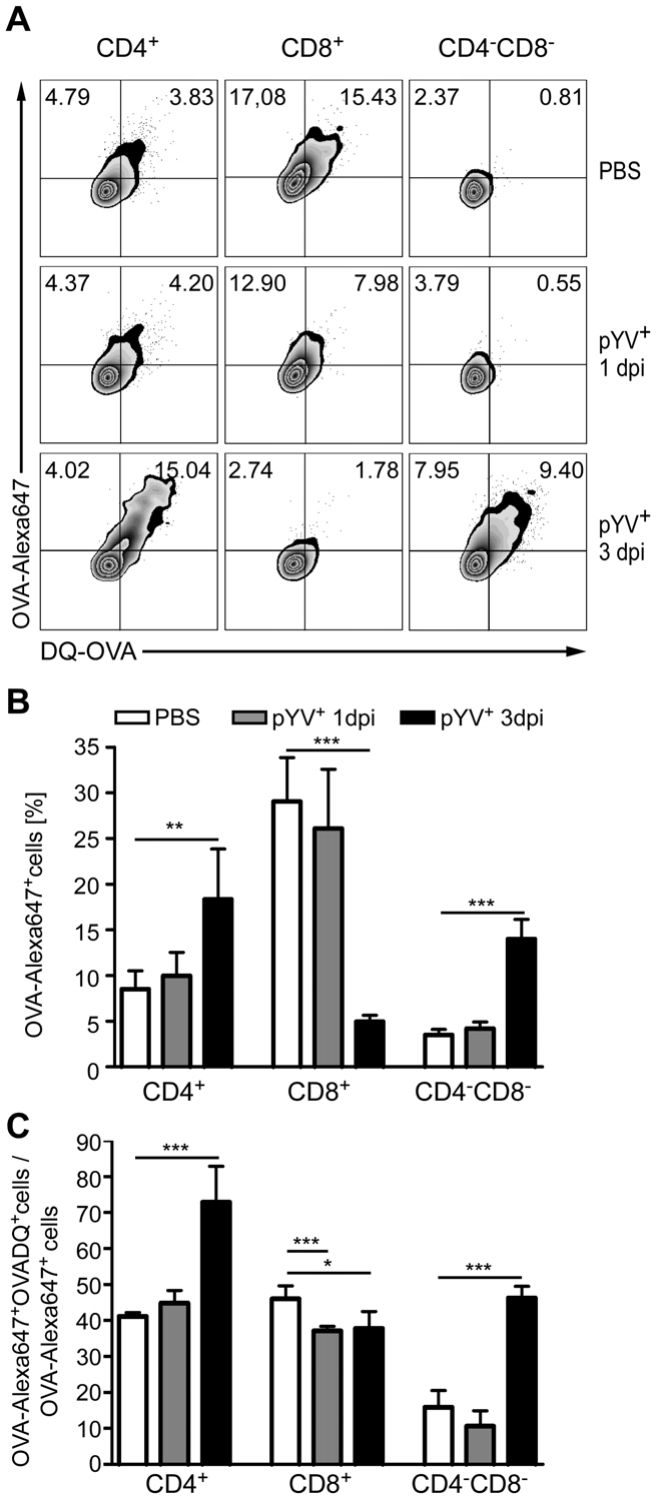
Analysis of antigen uptake and degradation by DCs from mice infected with Ye. (**A–C**) C57BL/6 mice were injected i.v. with 5×10^4^ Ye pYV^+^ or PBS. 24 h or three days p.i. OVA-AlexaFluor647 and DQ-OVA was administered i.v.. Uptake of OVA-AlexaFluor647 and degradation of DQ-OVA by DC subpopulations in the spleen was analyzed 1 h later by flow cytometry. (**A**) Dot plots show OVA-AlexaFluor647^+^ and DQ-OVA^+^ DC subpopulations from the mice as indicated. The percentages of cells in the upper two quadrants are indicated. (**B**) The diagram shows the percentage of OVA-AlexaFluor647^+^ DC subpopulations as analyzed in (A, upper two quadrants). (**C**) DQ-OVA degradation by DC subpopulations was analyzed as the percentage of cells in the upper right quadrant/percentage of cells in the two upper quadrants x 100% as shown in A. The data are representative of 2 experiments with 5 mice per group. * indicates statistically significant differences.

To further elucidate whether antigen uptake and/or degradation by DC subpopulations is modulated by Ye *in vivo*, mice were co-injected i.v. 24 h or three days p.i. with AlexaFluor647-labeled OVA protein to analyze OVA uptake, and DQ-OVA, a self-quenched fluorescently-labeled OVA protein, which emits fluorescence upon proteolytic degradation in endosomes and lysosomes [41]. OVA uptake and degradation was analyzed 1 h post OVA administration by flow cytometry. In line with previous data [20], CD8α^+^ DCs displayed a higher uptake of OVA-Alexa647 (Fig. 2A) than CD4^+^ or CD4^−^CD8α^−^ DCs (Fig. 2A and B). 24 h and three days p.i. with Ye, we observed 20% or 80% less OVA-Alexa647^+^CD8α^+^ DCs, respectively, compared to PBS-treated mice. The percentage of OVA-Alexa647^+^CD4^+^ and CD4^−^CD8α^−^ DCs 24 h post Ye infection was similar to that of PBS-treated mice (Fig. 2A and B). Interestingly, we found that the percentage of OVA-Alexa647^+^CD4^+^ and CD4^−^CD8α^−^ DCs was increased 1,5- to 4-fold three days p.i. with Ye, respectively (Fig. 2A and B).

Furthermore, we determined antigen degradation of DC subpopulations *in vivo* by analyzing the percentage of OVA-Alexa647^+^DQ-OVA^+^ cells related to all cells, which took up OVA-Alexa647 after infection of mice with Ye (Fig. 2A upper right quadrant/upper two quadrants). Overall, 40% of CD4^+^ and CD8α^+^ DCs from PBS-treated mice degraded OVA, in contrast to only 15% of CD4^−^CD8α^−^ DCs (Fig. 2C). 24 h and three days p.i. of mice with Ye, OVA degradation by CD8α^+^ DCs was slightly but significantly reduced by 15% compared to PBS-treated mice (Fig. 2C). CD4^+^ and CD4^−^CD8α^−^ DCs showed similar OVA degradation as PBS-treated mice 24 h post infection. In contrast, three days post Ye infection 70% of all OVA-Alexa647^+^CD4^+^ were also positive for DQ-OVA, indicating an increase in OVA degradation. Analyzing the CD4^−^CD8α^−^ DCs we observed, similar to CD4^+^ DCs, an increase in OVA degradation from 15% to 45%.

Altogether, these data indicate that Ye specifically reduces antigen uptake and degradation by CD8α^+^ DCs, while CD4^+^ and CD4^−^CD8α^−^ DCs showed an increase in OVA uptake and degradation three days p.i. with Ye.

### Ye induces maturation in DC subpopulations *in vivo*

**Figure 3:**
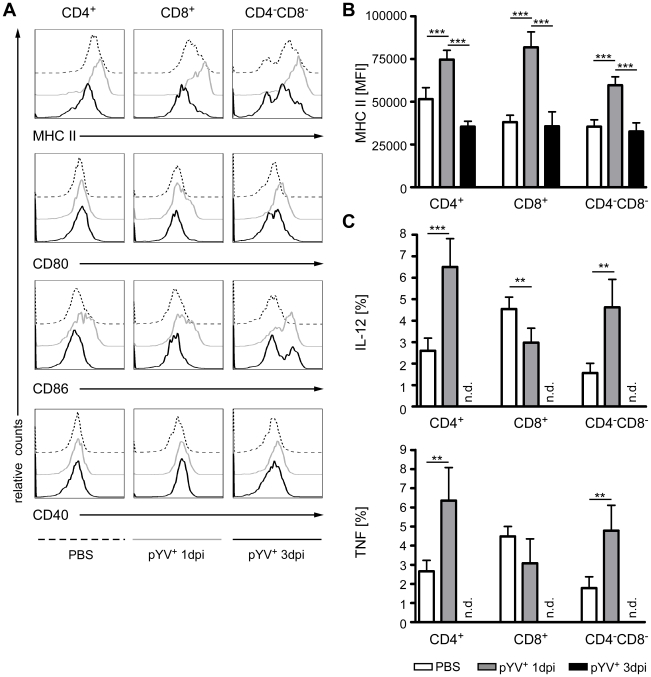
Analysis of DC maturation and cytokine production in mice infected with Ye. (**A–C**) C57BL/6 mice were injected i.v. with 5×10^4^ Ye pYV^+^ or PBS. 24 h or three days p.i. the expression of MHC class II, CD86, CD80, and CD40 molecules or the production of intracellular cytokines by DC subpopulations in the spleen was analyzed by flow cytometry. (**A**) Histograms show expression of the indicated molecules of 7-AAD^−^ DC subpopulations. The diagrams show the mean fluorescence intensity of MHC class II expression (**B**) or the percentage of each DC subset producing IL-12 or TNF (**C**). Data are representative of 2 experiments with 5 mice per group. * indicates statistically significant differences. n.d. not determined.

As maturation of DCs is induced upon the encounter of danger signals such as LPS and is essential for T-cell activation, we analyzed the induction of maturation of DC subpopulations by Ye upon *in vivo* infection. Mice were infected with Ye and spleen cells were subjected to flow cytometry analysis of MHC class II and the costimulatory molecules CD80, CD86 and CD40 (Fig. 3A). The expression of MHC class II molecules was up-regulated up to two-fold 24 h p.i. in all three DC subsets. However, expression of MHC class II molecules was significantly reduced three days p.i. compared to 24 h p.i. (Fig. 3B). Similar results were found for CD8α^+^ and CD4^−^CD8α^−^ DCs in terms of CD80, CD86, and CD40 expression, and for CD4^+^ DCs in terms of CD86 expression. In contrast, no up-regulation of CD40 and only a slight up-regulation of CD80 was observed for CD4^+^ DCs upon Ye infection (Fig. 3A). Taken together, Ye induces up-regulation of maturation markers in all splenic cDC subsets 24 h post infection, but this up-regulation is absent three days post infection.

Consistently, infection of mice for 24 h with Ye resulted in the increased production of the proinflammatory cytokines IL-12 and TNF (Fig. 3C) by CD4^+^ and CD4^−^CD8α^−^ DCs compared to PBS-treated mice. Interestingly, CD8α^+^ DCs produced less IL-12 and TNF upon *Yersinia* infection (Fig. 3C), indicating that Ye specifically targets this cDC subset. In summary, Ye differentially affects splenic DC subpopulations in terms of maturation and cytokine production.

### Ye injects bacterial proteins into splenic DCs

**Figure 4:**
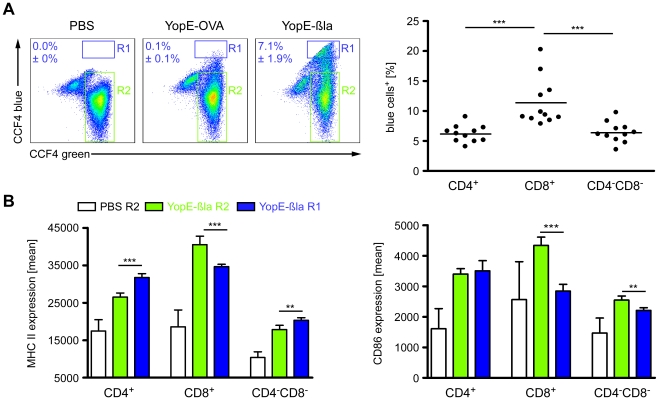
Injection of Yops into DCs upon infection of mice with Ye. (**A & B**) C57BL/6 mice were injected i.v. with 5×10^5^ Ye E40-YopE53β-lactamase (YopE-βla), 5×10^5^ Ye E40-YopE53-OVA (YopE-OVA), or PBS. 24 h p.i. the percentage of DC subpopulations in the spleen injected with YopE-βla was analyzed by flow cytometry using CCF4 substrate emitting a blue fluorescence when degraded by β-lactamase in the cytosol. (**A**) The dot plots show the percentage ± standard deviation of CCF4-blue^+^CD11c^hi^ cells from mice treated with PBS, infected with the negative control Ye YopE-OVA mutant strain, or infected with the Ye YopE-βla mutant strain. R1 indicates the blue cells (Yop injected) and R2 the green cells (not injected). The diagram shows the percentage of blue^+^ DC subpopulations of mice infected with the Ye YopE-βla mutant strain. Data shown are the summary of 2 independent experiments with 5–6 mice infected with YopE-βla mutant strain in each experiment. (**B**) The expression of MHC class II and CD86 on DC subpopulations from mice injected with PBS or infected with YopE-βla mutant strain was analyzed as described in (A) (R1: blue cells, R2: green cells). The diagrams show the MHC class II or CD86 expression of the DC subpopulations as mean fluorescence and are representative for 2 independent experiments with 5–6 mice infected with YopE-βla mutant strain in each experiment. * indicate statistically significant differences.

Yops are the major pathogenicity factors of Ye and are injected into host cells via the TTSS. By this means, Ye inhibits phagocytosis, antigen presentation and both NF-κB and MAPK signaling cascades [44]. Köberle et al. and others revealed that Ye, *Y. pseudotuberculosis* and *Y. pestis* preferentially target macrophages, DCs, and Gr-1^+^ cells [45]–[47]. To analyze whether Ye preferentially targets a splenic DC subpopulation, we adapted a recently described, new reporter system for monitoring injection of bacterial proteins into host cells via the TTSS [45], [48], [49]. We used Ye wild type strain expressing either a YopE-β-lactamase fusion protein or YopE-OVA as control for mouse infection experiments. Spleen cells were stained with the lipophilic CCF4-AM [50], an esterified form of the CCF4 substrate. After entry into living cells (Figure S1), cytoplasmic esterases rapidly convert CCF4-AM into negatively charged CCF4, which is retained in the cytoplasm. Excitation of CCF4 cumarin residue at 409 nm results in a fluorescence resonance energy transfer (FRET) to the fluorescein residue, leading to emission of green fluorescence at 520 nm. Cleavage of CCF4 substrate by β-lactamase interrupts FRET leading to light emission at 447 nm (blue fluorescence). Therefore, injection of YopE-β-lactamase by Ye into DCs can be determined by analyzing the CCF4-green^+^blue^+^ DCs by flow cytometry. 24 h p.i. with Ye YopE-β-lactamase mutant strain, 7.1±1.9% CD11c^hi^ cells displayed blue fluorescence (Fig. 4A: R1 in dot plots), indicating injection of the YopE-β-lactamase fusion protein into the cells. No β-lactamase^+^ cells (R1) and only 0.1±0.1% β-lactamase^+^ cells (R1) could be detected in mice either treated with PBS or infected with the control Ye mutant strain YopE-OVA, respectively. Flow cytometry analysis of the different DC subpopulations for injection of the YopE-β-lactamase revealed that significantly more CD8α^+^ DCs (11.4±1.2%) were Yop injected compared to CD4^+^ (6.2±0.4%) and CD4^−^CD8α^−^ DCs (6.4±0.5%) 24 h p.i. (Fig. 4 A).

To further elucidate direct effects of Yop-injection into the DC subsets we analyzed the β-lactamase^+^ (R1 in Fig. 4A) and β-lactamase^−^ DC subpopulations (R2 in Fig. 4A) from mice infected with the Ye E40 YopE-β-lactamase mutant strain for their expression of MHC class II and CD86. Overall, the expression of these markers by all DC subpopulations from Ye E40 YopE-β-lactamase mutant strain-infected mice (R1 as well as R2) was increased compared to DCs from uninfected control mice (Fig. 4B). These results are similar to that from mice infected with Ye WA-314 pYV^+^ wild type strain (Fig. 3A & B) used in all other experiments of this study. The expression of MHC class II was higher (by 15%) in β-lactamase^+^CD4^+^ and β-lactamase^+^CD4^−^CD8α^−^ DCs compared to the β-lactamase^−^ cells, while the MHC class II expression of β-lactamase^+^CD8α^+^ DCs was 15% less than that of β-lactamase^−^CD8α^+^ DCs (Fig. 4B). In contrast, CD86 expression of β-lactamase^+^CD8α^+^ and CD4^−^CD8α^−^ DCs was reduced by 40% or 15%, respectively, compared to the corresponding β-lactamase^−^ cells. CD86 expression by CD4^+^ DCs was independent of Yop-injection.

In conclusion, Ye directly targets a significant fraction of splenic DCs, most frequently the CD8α^+^ subpopulation, leading to less expression of MHC class II and CD86 by these Yop-injected cells when compared to the Yop-uninjected cells.

### Ye induces loss of splenic DC subpopulations

**Figure 5:**
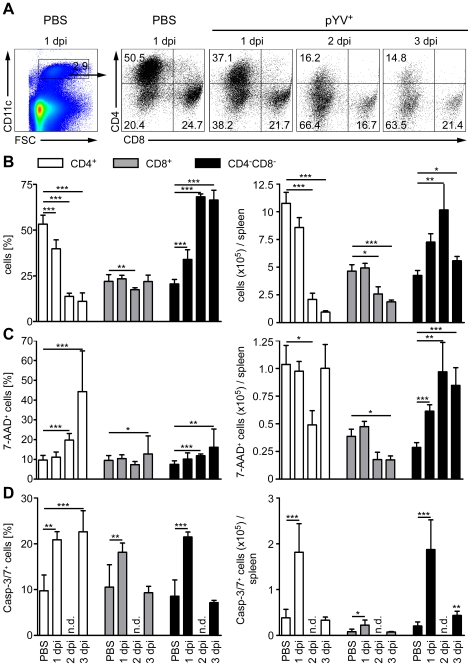
Analysis of DC subpopulations in mice infected with Ye. (**A–D**) C57BL/6 mice were injected i.v. with 5×10^4^ Ye pYV^+^ or PBS. (**A & B**) 24 h to three days p.i. the splenic DC subpopulations stained with antibodies against CD11c, CD4, and CD8 followed by 7-AAD staining were analyzed by flow cytometry. (**A**) Dot plots show the gating strategy and the percentages of the viable DC subpopulations in the spleen during infection of mice with Ye. The data are representative for 5 experiments with 5 mice per group. (**B**) The diagrams show the percentage (left diagram) and the total numbers (right diagram) of the indicated DC subpopulations per spleen during infection of mice with Ye. The data are the summary results of two to 5 experiments with 4–5 mice per group. (**C**) 24 h to three days p.i. the percentage (left diagram) and the total numbers (right diagram) of 7-AAD^+^ DC subpopulations per spleen were analyzed by flow cytometry. The data are the summary results of two to 5 experiments with 4–5 mice per group. (**D**) 24 h and three days p.i. the percentage (left diagram) and the total numbers (right diagram) of caspases-3/7^+^ DC subpopulations per spleen were analyzed by flow cytometry. n.d. not determined. Data are representative of 2 independent experiments with 5 mice per group. * indicate statistically significant differences.

**Figure 6:**
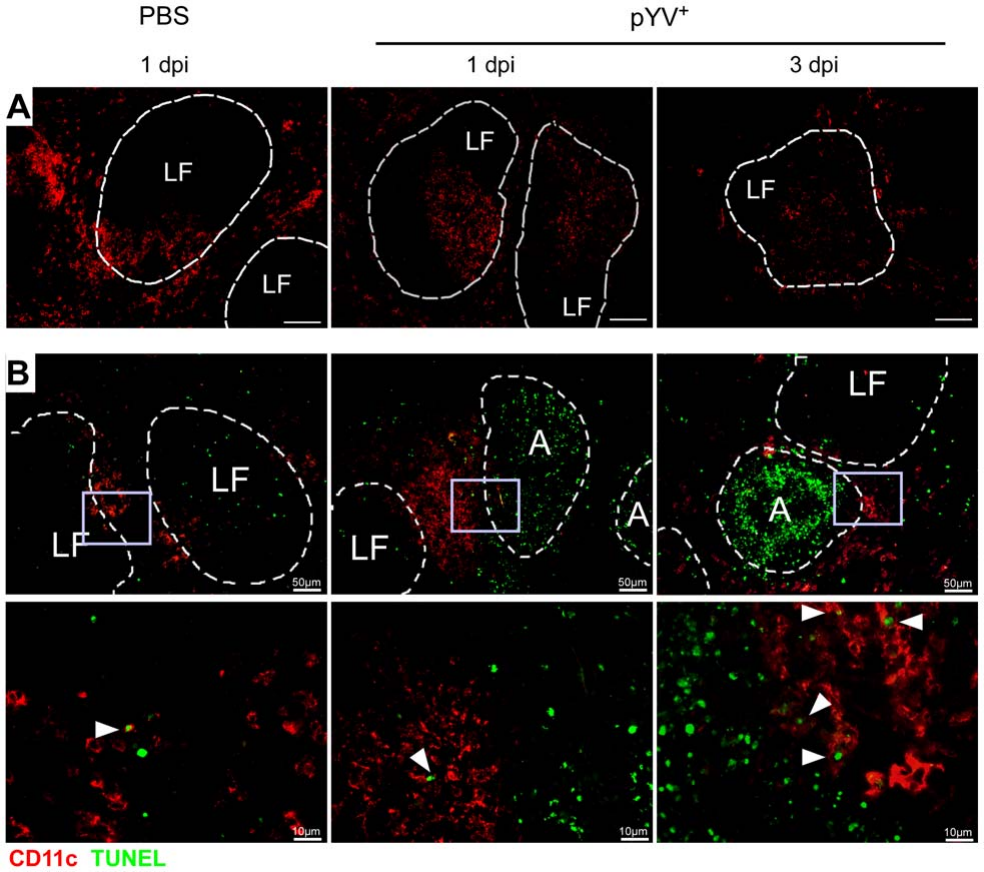
Immunofluorescence analysis of DCs from mice infected with Ye. C57BL/6 mice were injected i.v. with 5×10^4^ Ye pYV^+^ or PBS. (**A**) Cryosections of spleen from mice treated with PBS or Ye were stained for CD11c (red), Bar  = 100 µm. (**B**) Cryosections of spleen from mice treated with PBS or Ye were stained for CD11c (red, surface staining) and TUNEL (green, nuclear staining). Arrowheads indicate double positive cells, A abscesses, LF lymphoid follicle.

Mice treated with LPS or infected with *E. coli* showed a transient loss of CD4^+^ and CD8α^+^ DCs in the spleen by apoptosis [51]. Here, apoptosis was mediated via TLR4 and TRIF signaling. To elucidate whether Ye induces DC death *in vivo*, the number of live and dead splenic DC subpopulations from Ye-infected and PBS-treated mice were analyzed (Fig. 5). Upon infection with Ye CD4^+^ (90%) and to a lesser extent CD8α^+^ DCs (65%) were lost (Fig. 5A and B). Specifically, the number of CD4^+^ and CD8α^+^ DCs declined from 1.1×10^6^ cells and 4.7×10^5^/spleen in PBS-treated mice to 1×10^5^ cells and 1.7×10^5^/spleen three days p.i. with Ye, respectively (Fig. 5B). In contrast, the number of CD4^−^CD8α^−^ DCs in the spleen increased upon Ye infection compared to that in control mice. In addition, we did immunofluorescence microscopy of cryosections from the spleen of Ye or PBS-treated mice analyzing the CD11c^+^ cells. Ye infection leads to the migration of CD11c^+^ cells from the marginal zone (PBS-treated mice) to the T-cell zone of the lymphoid follicles and to a reduced number of CD11c^+^ cells three days p.i. (Fig. 6A). CD4 or CD8α down-regulation on CD4^+^ or CD8α^+^ DCs was not observed upon infection of splenocytes with Ye *in vitro* (Fig. S2), indicating that the loss of DC subpopulations is not due to simple down-regulation of these receptors.

We next examined whether the loss of CD4^+^ and CD8α^+^ DCs is due to the induction of cell death upon Ye infection. Therefore, we gated on 7-AAD^+^ DC subpopulations because 7-AAD is taken up by cells with a damaged cell membrane (gating strategy shown in Fig. S3A and S3B). The percentage of 7-AAD^+^CD4^+^ DCs increased from 10% in PBS-treated mice to 44% upon infection with Ye (Fig. 5C left diagram), whereas the total number of 1.0×10^5^ 7-AAD^+^CD4^+^ DCs observed in PBS-treated mice was not increased upon Ye infection (Fig. 5C right diagram). This can be explained by the decreased number of CD4^+^ DCs two and three days post Ye infection. In line with these data, CD4^+^ DCs displayed an increase in active caspases-3/7^+^ cells of 20% upon Ye infection, compared to 10% in PBS-treated mice (Fig. 5D left diagram; gating strategy shown in Fig. S3A and S3C). Thus, a significant increase in the number of caspases-3/7^+^CD4^+^ DCs could be observed 24 h post infection. Cell death analyses of CD8α^+^ DCs revealed no increase of 7-AAD^+^, but a slight increase of caspases-3/7^+^ DCs 24 h post Ye infection, compared to PBS-treated mice. Ye induced cell death in CD4^−^CD8α^−^ DCs as shown by the increase of the percentage and absolute numbers of 7-AAD^+^ and caspases-3/7^+^ cells.

Furthermore, we used TUNEL staining for cell death detection and found a massive increase of TUNEL^+^ cells located in *Yersinia*-induced abscesses. However, only very few single apoptotic DCs (CD11c^+^TUNEL^+^) were found in *Yersinia*-induced abscesses in the spleen of infected mice (Fig. 6B), and total numbers of 7-AAD^+^ CD4^+^ and CD8α^+^ DCs were unaltered upon Ye infection and in PBS-treated control mice.

Hence, Ye-induced apoptosis of CD4^−^CD8α^−^ DCs results in a rapid recruitment of the exact same DC subtype during infection as the population size does not decline. We also observed an increase in the percentage, but not in total numbers, of cell death in CD4^+^ and CD8α^+^ DCs; thus, the Ye-induced loss of CD4^+^ and CD8α^+^ DCs in the spleen can only partly be explained by the induction of apoptosis in these splenic DC subpopulations.

### Ye-induced loss of CD4^+^ and CD8α^+^ DCs is mediated via TLR4 and TRIF signaling

**Figure 7:**
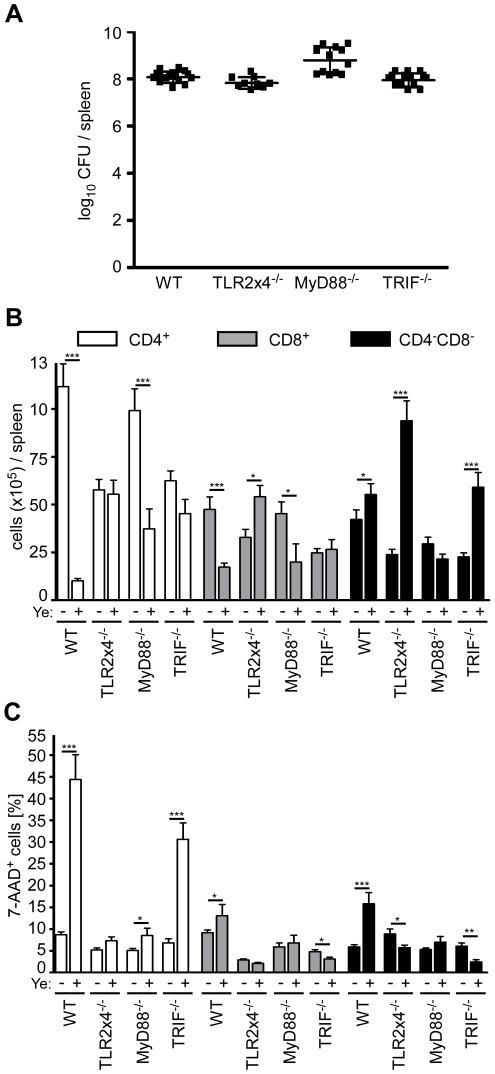
Analysis of DC subpopulations in TLR2^−/−^x4^−/−^, MyD88^−/−^, and TRIF^−/−^ mice infected with Ye. (**A–C**) Mice were injected i.v. with Ye as follows to achieve a similar bacterial load per spleen: C57BL/6 and TRIF^−/−^ with 5×10^4^ pYV^+^, TLR2^−/−^x4^−/−^ with 5×10^3^ pYV^+^, and MyD88^−/−^ with 5×10^2^ pYV^+^, or PBS. The diagrams show the bacterial load as log_10_ colony forming units (CFU) (**A**), the total numbers of DC subpopulations (**B**) and the percentage (**C**) of 7-AAD^+^ DC subpopulations per spleen three days p.i. with Ye of the indicated mouse strains. The data show the summary results from three independent experiments with three to 5 mice per group in each experiment. * indicate statistically significant differences.

To elucidate whether the loss of CD4^+^ and CD8α^+^ DCs induced by Ye is due to TLR signaling, we analyzed the number of live and dead DC subpopulations in the spleen from *TLR2^−/−^xTLR4^−/−^*, *MyD88^−/−^*, and *TRIF^−/−^* mice. Mice were infected with different doses of Ye to obtain similar bacterial burden in the spleen three days p.i. (Fig. 7A). Similar to C57BL/6 wild type mice, the number of total viable CD4^+^ and CD8α^+^ DCs in *MyD88^−/−^* mice decreased up to 80% upon Ye infection (Fig. 7B), whereas more or less similar numbers of these subpopulations could be observed in *TLR2^−/−^xTLR4^−/−^* and *TRIF^−/−^* mice. Furthermore, the number of CD4^−^CD8α^−^ DCs per spleen was unchanged upon infection in wild type and *MyD88^−/−^* mice, but was significantly increased in *TLR2^−/−^xTLR4^−/−^* and *TRIF^−/−^* mice. Hence, Ye-induced loss of CD4^+^ and CD8α^+^ DCs in wild type mice is mediated via signaling through TLR4 and TRIF.

Upon Ye infection a dramatic increase of 7-AAD^+^CD4^+^ DCs was observed in wild type and *TRIF^−/−^* mice, but not in *TLR2^−/−^xTLR4^−/−^* and *MyD88^−/^* ^−^ mice (Fig. 7C), indicating that the induction of cell death in CD4^+^ DCs by Ye is mediated via MyD88 signaling. Ye induces a decrease of CD4^+^ DCs by 60% in *MyD88^−/−^* mice, while the cell death rate of this population is less than 10%, indicating that induction of cell death seems not to be responsible for the observed decrease of CD4^+^ DCs.

### Increased proliferation and inhibition of *de novo* generation of CD4^+^ DCs upon Ye infection

**Figure 8:**
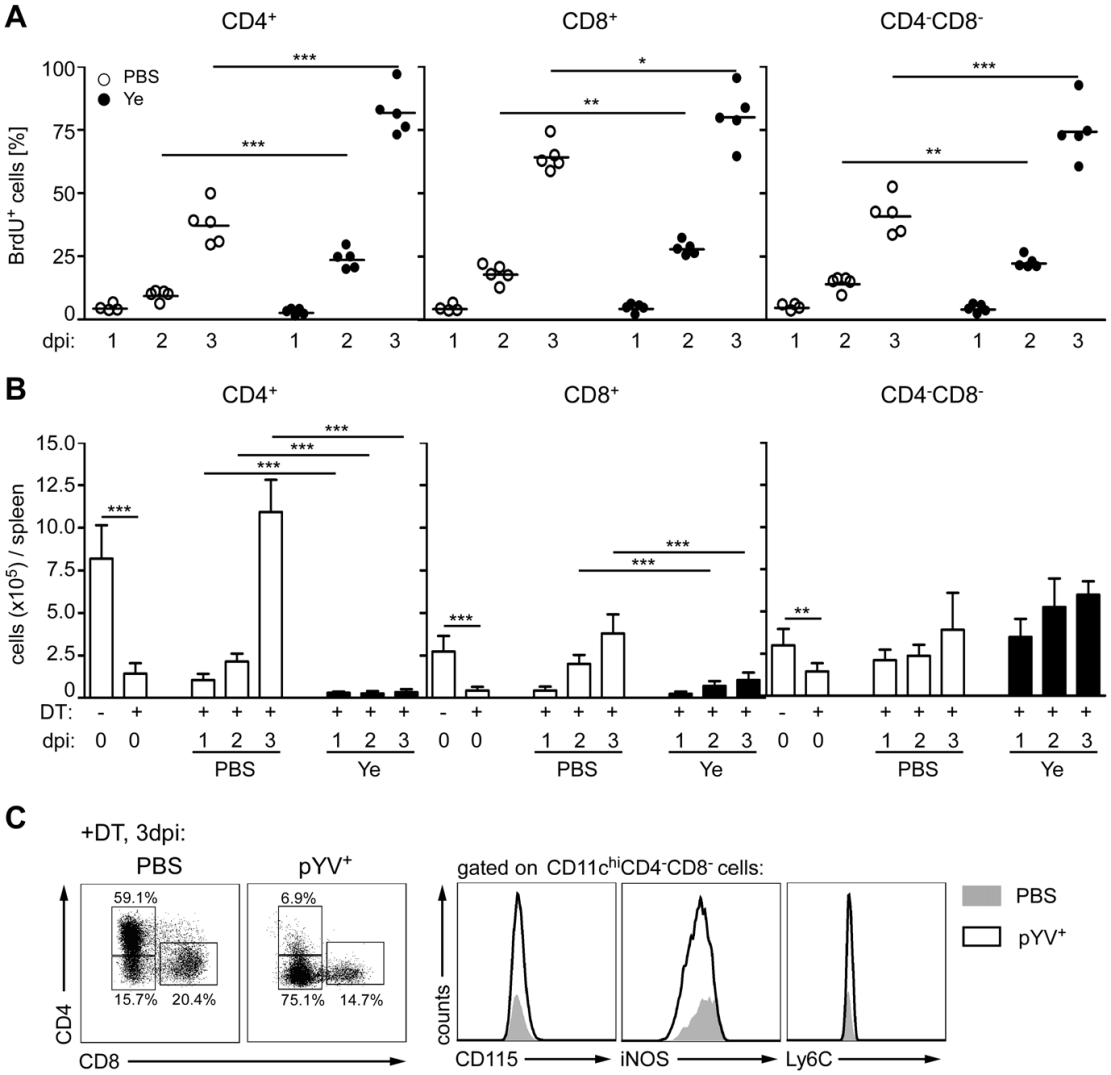
Proliferation and recovery of DC subpopulations from mice infected with Ye. (**A**) C57BL/6 mice were injected i.v. with 5×10^4^ Ye pYV^+^ or PBS and daily with BrdU. The percentage of BrdU^+^ DC subpopulations was analyzed one to three days p.i. by flow cytometry. Data are representative of 2 independent experiments with 5 mice per group. (**B**) CD11c.DOG mice were once injected i.p. with diphteria toxin (DT) to ablate CD11c^hi^ cells. 24 h later the mice were injected i.v. with 5×10^4^ Ye pYV^+^ or PBS. The absolute numbers of DC subpopulations in the spleen were analyzed by flow cytometry one to three days p.i.. The data show the summary results from two independent experiments with 4–5 mice per group in each experiment. (**C**) CD11c.DOG mice were treated as described in (B). The CD4^−^CD8α^−^ DCs were analyzed three days p.i. for the indicated antigens characterizing inflammatory DCs. * indicate statistically significant differences.

To determine whether Ye modulates the proliferation capacity of DCs, BrdU incorporation into DC subpopulations from C57BL/6 mice infected with Ye or treated with PBS was analyzed by flow cytometry. As described by others [52], [53] CD8α^+^ DCs showed a higher BrdU incorporation than CD4^−^CD8α^−^ and CD4^+^ DCs (Fig. 8A). Two and three days p.i. with Ye the percentage of BrdU^+^ DC subpopulations was significantly increased compared to PBS-treated mice. In fact, 81.7±4.1% BrdU^+^CD4^+^ DCs could be observed three days p.i. compared to 37.1±3.6% in PBS-treated mice. BrdU^+^ CD4^−^CD8α^−^ and CD8α^+^ DCs increased from 40.8±3.4% to 74.3±5.1% and 64.2±2.6% to 80.1±5.0% 3 days p.i., respectively. In conclusion, Ye induced a faster proliferation of all DC subpopulations.

All splenic DCs examined in this study originate from the same precursors. In the bone marrow myeloid precursors (MPs) differentiate into macrophage and DC precursors (MDPs). They develop into common DC progenitors (CDPs) and then pre-DCs, which give exclusively rise to cDCs [54], [55]. To analyze whether the *de novo* generation of the DC subpopulations in the spleen is impaired by Ye we used a transgenic mouse model, in which the DC subpopulations, but not their precursors, were ablated by diphtheria toxin (DT) treatment [56]. To this end CD11c.DOG mice were injected once with DT one day prior to Ye infection, and the number of DC subpopulations was analyzed one to three days p.i.. In uninfected CD11c.DOG control mice DT treatment led to 84%, 82%, and 50% reduced numbers of CD4^+^, CD8α^+^, and CD4^−^CD8α^−^ DCs one day after DT treatment, respectively (Fig. 8B). Four days after DT treatment the number of all DC subpopulations increased to normal numbers, indicating that all DCs were recovered in the spleen at this time point. In contrast, in Ye-infected DT-treated CD11c.DOG mice, the number of CD4^+^ DCs did not recover during the period of observation; in fact, the number of CD4^+^ DCs one to three days p.i. was even less than that of CD11c.DOG mice after DT treatment, indicating that Ye blocks the *de novo* generation of CD4^+^ DCs. Likewise, the number of CD8α^+^ DCs did not completely recover after Ye infection compared to PBS-treated mice, whereas the number of CD4^−^CD8α^−^ DCs was even increased one to three days p.i. compared to PBS-treated mice, indicating that Ye infection leads to an increased *de novo* generation of CD4^−^CD8α^−^ DCs. To exclude the possibility that these CD4^−^CD8α^−^ DCs were inflammatory DCs generated from monocytes or monocyte precursors, CD4^−^CD8α^−^ DCs from DT-treated CD11c.DOG mice were analyzed for monocytic markers three days p.i. with Ye or treatment with PBS (Fig. 8C). The recovered CD4^−^CD8α^−^ DCs from both Ye-infected as well as PBS-treated mice did not express either iNOS, CD115 or Ly6C. These markers are characteristically expressed by TNF and iNOS producing DCs (Tip-DCs) or inflammatory macrophages but not by CD4^−^CD8α^−^ DCs [54]. Taken together, Ye stimulates increased proliferation of DCs, but higher cell death rates combined with inhibition of *de novo* generation lead to loss of CD4^+^ and CD8α^+^ DC subsets upon infection.

## Discussion

Strong efforts were made to elucidate the function of different splenic DC subsets, but little is known about their roles during bacterial infections. In previous work, we demonstrated that CD4^+^ T cells are essential in controlling infection by the extracellular bacterial pathogen Ye in mice [34], [35] and that Ye modulates DC functions of BM-DCs *in vitro*, leading to inhibition of T-cell proliferation [27], [40], [41]. However, infection experiments with BM-DCs can not be used to extrapolate the events operating upon infection of DCs *in vivo* because here, distinct DC subsets with different tissue distribution and functions exist. This study was aimed to investigate the effects of Ye infection on splenic DC subsets with respect to antigen uptake, antigen processing, cytokine production and subsequently T-cell proliferation *in vivo*.

Strikingly, we found an impaired ability to induce T-cell proliferation by CD8α^+^ but not by CD4^+^ or CD4^−^CD8α^−^ DCs upon Ye infection *in vivo.* This is due to a reduction of antigen uptake and degradation, as well as an inhibition of proinflammatory cytokine secretion. Adoptive transfer experiments with antigen-pulsed CD8α^+^ DCs demonstrated that they promote Th1 responses, whereas CD8α^−^ DCs promote Th2 responses [18], [57], [58]. Therefore, targeting of CD8α^+^ DCs by Ye may result in immune evasion of the pathogen as infection control requires CD4^+^ Th1 host responses [34], [37]. IL-12 is the key cytokine responsible for Th1 activation. CD8α^+^ DCs have been shown to produce high levels of IL-12 *in vivo* upon microbial stimulation or infection [57], [59]–[63]. On the other hand, CD8α^−^ DCs were also shown to produce IL-12 in response to TLR7 ligands [64] and LPS in the presence of anti-IL-10 [59]. Upon infection with Ye, IL-12 as well as TNF production was induced in CD4^+^ and CD4^−^CD8α^−^, but decreased in CD8α^+^ DCs, which in turn may account for reduced T-cell priming by CD8α^+^ DCs.

Macropinocytosis of fluid phase antigen is shown to be down regulated during DC maturation [65]–[67]. Ye infection led to a reduction in OVA uptake by CD8α^+^ DCs, which could be either due to Yop translocation or induction of DC maturation. Several facts argue against the second possibility: all DC subpopulations display largely mature phenotypes 24 h p.i. but only the CD8α^+^ DCs show reduced OVA uptake. All DC subpopulations display immature phenotypes 72 h p.i.. At this point OVA uptake by CD8α^+^ DCs was significantly reduced, but increased in CD4^+^ and CD4^−^CD8α^−^ DCs. As CD8α^+^ DCs are considered to be the most effective splenic DC-type in terms of antigen uptake *in vivo* [20], the findings suggest that CD8α^+^ DCs are specifically targeted by Ye. Consistently, OVA uptake was shown to be mediated predominantly via the macrophage-mannose receptor (MR). This process is independent of DC maturation [68], [69]. The MR is expressed only by splenic CD8α^+^ DCs [68], [69] explaining the 3,5–7 fold higher OVA uptake by CD8α^+^ DCs compared to CD4^+^ and CD4^−^CD8α^−^ DCs in control mice. Previous studies with BM-DCs showed, that Ye YopP inhibits OVA uptake via receptor-mediated endocytosis, suggesting a direct effect of Ye on OVA uptake by CD8α^+^ DCs [40]. Whether Ye infection causes a down regulation of MR is unknown.

DC maturation is crucial for their ability to activate T cells. Here we found that Ye rapidly induced DC maturation in all DC subsets, which was absent three days p.i.. This could be due to the down-regulation of the maturation markers or a rapid turnover of all DC subsets induced by Ye. *In vitro* infection of BM-DCs with Ye revealed an inhibition of DC maturation with respect to expression of MHC II and costimulatory molecules as well as proinflammatory cytokine production [27]. These *in vitro* data differ from the results in this study, but can be explained by a lesser Yop-injection rate into DCs *in vivo*. Usually, up-regulation of maturation markers is observed upon bacterial infections at different time points, but their down-regulation is not described [70]–[73]. De Trez et al. showed that infection of mice with live or heat-killed apathogenic *E. coli* induced maturation of splenic DCs 6 to 9 h p.i. in a TLR4 and TRIF signaling dependent manner [51]. In the present study, application of heat-killed Ye did not induce maturation of splenic DCs (data not shown), suggesting that effects other than TLR activation are responsible for DC maturation in *Yersinia* infection. In *Salmonella*-infected mice, maturation is induced directly in DCs associated with intracellular *Salmonella* and indirectly via *Salmonella*-induced TNF production [71]. Whether TNF-induced maturation plays a role in Ye-induced DC maturation has to be further elucidated. In conclusion, maturation of DCs *in vivo* is variably regulated in response to different bacterial pathogens.

We and others could show that Ye, *Y. pestis* and *Y. pseudotuberculosis* preferentially target professional phagocytes like Gr-1^+^ cells, macrophages, and DCs *in vivo* by using a β-lactamase reporter system to detect Yop injection into host cells [45]–[47]. Ye targets specifically CD8α^+^ DCs, which in turn express less MHC class II and CD86 than non-injected CD8α^+^ DCs. This implies that Ye induces DC maturation which is then reversed by Yop injection. In contrast, Yop-injected B cells displayed a significantly increased expression of CD69 compared to non-Yop-injected B cells, indicating activation of these cells by Ye [46]. The fact that CD8α^+^ DCs are targeted most frequently by Ye is in agreement with their impaired OVA uptake and degradation, and cytokine production leading to less T cell activation. It was shown that YopH specifically inhibits both T and B cell activation *in vitro* [74], [75]. Whether Yops secreted into T cells have a direct effect on T-cell proliferation *in vivo* remains to be shown. It also remains to be determined why Yops are specifically injected into CD8α^+^ DCs and how this affects DC functions like cytokine production and T cell priming *in vivo*. The DC subsets may differ in their ability to chemotax towards bacteria. Moreover, as binding of Ye to β1-integrins is essential for Yop injection [46] the expression of β1-integrins by DC subsets, could result in the selection for a specific DC subset.

The reduction in CD4^+^ and CD8α^+^ DCs in the spleen upon Ye infection is dependent on TLR4-TRIF signaling. Upon infection of mice with *E. coli* or treatment with LPS, the number of CD4^+^ and CD8α^+^ DCs was markedly reduced 48 hours later [15], [51], a result of TLR4-TRIF signaling induced apoptosis [51]. Recent findings demonstrate that Type I IFN regulate the turnover of splenic conventional DCs *in vivo* [76]. Whether Type I IFNs are involved in the turnover of conventional DCs upon Ye infection was not analyzed. Furthermore, oral infection of mice with *Salmonella typhimurium* specifically induces death of CD8α^+^ DCs, but not of CD8α^−^ DCs, via MyD88 and TNFR1 signaling [77]. However, Ye induced death of predominantly CD4^+^ DCs in a MyD88 dependent manner. Recently it was shown that *Y. pseudotuberculosis* induces caspase-1-dependent pyroptosis in activated macrophages *in vivo* [78]. Caspase-1 activation might be responsible for some of the loss of DCs upon Ye infection, particularly on day 3 p.i. when only a fraction of the 7-AAD^+^ cells are caspase-3/7^+^. Unfortunately, the β-lactamase reporter system can only be used in viable cells and the detection of Yop-injection into apoptotic cells *in vivo* is limited.

Here we show for the first time that a bacterial pathogen affects the homeostasis of specific DC subpopulations. It is likely that the increased proliferation in combination with the impaired *de novo* generation of CD4^+^ and CD8α^+^ DCs cause the Ye-induced loss of these DC subpopulations. Recently, Hochweller et al. could show that DC depletion led to enhanced DC generation as a result of increased differentiation rates from pro-DCs to pre-DCs and conventional DCs in the spleen [79]. This suggests, that the higher proliferation of all DC subpopulations upon Ye infection is due to an increased differentiation of precursor cells into conventional DCs. Upon infection of mice with *Listeria monocytogenes* or *Leishmania major* recruited inflammatory monocytes differentiate into Tip-DCs at sites of infection in a CCR2-dependent manner [80]–[82]. Interestingly, the observed increased numbers of CD4^−^CD8α^−^ DCs upon Ye infection do not represent Tip-DCs. Nevertheless, more detailed studies are needed to uncover the mechanism responsible for the disturbed CD4^+^ DC homeostasis upon Ye infection. Furthermore, it must be stressed that the observed changes in DC functions described herein occurred upon infection with high doses of bacteria; it might well be that the quantity of bacterial pathogenicity factors operating might be important for the observed effects.

In summary, this study demonstrates that Ye affects splenic DC subpopulations in different ways: Ye directly injects Yops into a portion of all DC subpopulations. In addition, infection of mice with Ye leads to a massive reduction of CD4^+^ DCs in a TLR4- and TRIF-dependent manner. Moreover, we cannot exclude that DC subpopulations are differentially susceptible or resistant to pathogenicity factors such as Yops of Ye. Nevertheless, the direct and indirect effects of Ye on DC subpopulations may contribute to reduced T-cell proliferation and thus immune evasion of the pathogen.

Our data and published reports (e.g. *Mycobacterium tuberculosis*, *Salmonella typhimurium*, *E. coli* [15], [51], [62], [83]) suggest that (i) splenic DC subsets are differentially affected by various bacterial pathogens, and (ii) primarily indirect effects exerted by systemic infection contribute to alterations of the majority of DCs *in vivo* rather than direct interactions of DCs with pathogens. This together with different functions of the DC subpopulations leads to the variability of DC responses to various bacterial infections.

## Materials and Methods

### Mice and infection

Ethics statement: Animal experiments were performed in strict accordance with the German regulations of the Society for Laboratory Animal Science *(GV-SOLAS)* and the European Health Law of the Federation of Laboratory Animal Science Associations (FELASA). The protocol was approved by the Regierungspräsidium Tübingen (Permit Number: IM5/08). All efforts were made to minimize suffering.

Female C57BL/6JolaHsd mice were purchased from Harlan Winkelmann (Borchen, Germany). *TLR2^−/−^xTLR4^−/−^*, *TRIF^−/−^* (LPS2) [84], *MyD88^−/−^* [85], OT-II [43], and CD11c.DOG [56] mice with a genetic C57BL/6 background were bred under specific pathogen-free conditions in the animal facilities of the University Clinic of Tübingen and the University of Tübingen. Mice used for experiments were between 6–9 weeks of age and were provided food and water *ad libitum*.

Mice were infected with the indicated amount of Ye WA-314 (serotype 0∶8) from frozen stock suspensions in 200 µl PBS into the tail vein. As a control, mice were infected only with 200 µl PBS. For the translocation experiments mice were infected with Ye 5×10^5^ E40-YopE53-βlactamase or E40-YopE53-OVA [46] as control one h after treatment of the mice with 2.5 mg desferoxamine mesylate salt (Sigma) in PBS i.v.. The bacterial load in the spleen was assessed after plating serial dilutions of the cell suspensions obtained on Müller-Hinton agar plates and was comparable for all mouse strains. For systemic DC depletion BAC transgenic CD11c.DOG mice, that express the human diphtheria toxin receptor under control of the CD11c promoter, were injected intraperitoneally with 8 ng/g bodyweight of diphteria toxin (Sigma) in PBS one day before Ye infection.

### Cell preparation

Spleens were cut into small pieces and then digested for 30 min at 37°C in 2 ml modified RPMI 1640 + 2% FCS medium containing collagenase (1 mg/ml; type IV; Sigma-Aldrich) and DNase I (150 µg/ml, Roche). To disrupt DC-T cell complexes, EDTA (0.1 ml, 0.1 M (pH 7.2)) was added, and mixing continued for 5 min. Single cell suspensions were made by pipetting the digested organs. Undigested fibrous material was removed by filtration and erythrocytes were lysed with lysis buffer (150 mM NH_4_Cl, 10 mM KHCO_3_, 2 mM NaEDTA). The total number of cells was determined by trypan blue exclusion. Spleen cells were prepared from individual mice for all experiments except those of Fig. 1B and 1C. In these experiments, CD11c-expressing cells were enriched from single cell suspensions of spleen pooled from 3 or 7 mice by MACS technology using N418 magnetic beads (Miltenyi Biotec) following the manufacturer's protocol. Cells were blocked and stained with CD11c-APC, CD4-PE, and CD8α-FITC in PBS. Then the DC subpopulations were sorted on a FACS Aria cell sorter (BD Biosciences), reanalyzed on a Canto-II flow cytometer and used for co-culture experiments *in vitro* (as described below).

### Flow cytometry

FACS buffer in PBS containing 1% FBS (Sigma-Aldrich) and 0.09% NaN_3_ (Sigma-Aldrich) was used for all incubations and washing steps. Before staining, cells were incubated for 15 min at 4°C with hybridoma supernatant from 2.4G2 cell line producing anti-FcgRII/III mAb. Cells were then stained with FITC, PE, APC, PE-Cy7, APC-Alexa700, pacific blue, or biotinylated conjugates of anti-CD11c (HL3, BD Biosciences, Miltenyi Biotec), CD8α (53-6.7, BD Biosciences), CD4 (RM4-5), CD3 (145.2C11), CD80 (16-10A1, BD Biosciences), CD86 (GL-1, BD Biosciences), CD40 (HM40-3, BD Biosciences), MHC II (M5/114.15.2, eBiosciences) for 20 min at 4°C. To exclude dead cells, 7-aminoactinomycin D (7-AAD; Sigma-Aldrich) was used in the case of surface staining and aqua life dead (Invitrogen) in the case of intracellular cytokine staining. When biotinylated mAb were used, cells were stained a second time with streptavidin-pacific orange or streptavidin-PE. To detect intracellular production of TNF, IL-6, IL-12, cells were seeded at 1×10^6^ cells/well in low adherence, 24-well plates (BD Falcon) and incubated for three to four h with brefeldin A (Biolegend) at a final concentration of 5 µg/ml. Cells were then stained for surface phenotype, fixed with 1% paraformaldehyde (Sigma-Aldrich) in PBS, permeabilized with 0,1% saponin (Sigma-Aldrich) and 0,5% BSA (Sigma-Aldrich) in PBS, and stained for intracellular cytokines with anti-TNF (MP6-XT22; BD Biosciences), IL-6 (MP5-20F3; Biolegend), IL-12p40 (C15.6; BD Biosciences) for 15 min at 4°C. Samples were acquired for 6 to 8-colour analysis using a Canto-II flow cytometer (BD Biosciences) with DIVA software (BD Biosciences) and further analyzed using FlowJo 7.5 software (TreeStar Inc). A total of 500,000–1,200,000 cells were acquired to ensure the analysis of at least 8,000 viable splenic DCs in each sample from individual mice.

For the detection of apoptotic DCs 3×10^6^ splenocytes were stained with FITC-Caspases 3 & 7 FLICA Apoptosis Dectection Kit (Immunohistochemistry Technologies, LLC) according to the manufacturer's instructions. Thereafter, cells were stained for cell surface antigens, measured and analyzed as described above.

For the detection of β-lactamase activity, surface molecule staining was performed as described above. Then 4×10^6^ cells were resuspended in 1x CCF4-AM staining solution supplemented with probenecid, prepared according to the manufacturer's instructions (Invitrogen). Following an incubation of 40 min at room temperature in the dark, cells were measured by flow cytometry on a Canto-II flow cytometer (BD Biosciences) and analyzed using FlowJo 7.5 software (Tree Star Inc).

For the detection of the BrdU labeling *in vivo* mice were daily injected i.v. with 1.0 mg BrdU (BD Biosciences). Surface molecule staining of splenocytes was performed as described above. Then the cells were fixed, permeabilized and BrdU-FITC staining was performed according to the manufacturer's protocol (BD Biosciences).

### T cell proliferation assays

CD4^+^ T cells were purified from spleen of OT-II mice as described previously [27] by using the CD4-negative T cell isolation kit and a magnetic-activated cell sorter system (Miltenyi Biotec). The resulting T cell preparations, containing 95 to 99% CD4^+^ T cells, were washed twice with ice-cold PBS, incubated with CFSE (5 µM; Invitrogen) in PBS for 5 min at room temperature, and then washed twice with ice-cold heat-inactivated FBS.

For the *in vivo* proliferation assays, 2×10^6^ CFSE-labeled T cells per mouse were adoptively transferred into the tail vain 6–8 h p.i. with Ye, followed by i.v. injection of ovalbumin protein (500 µg/mouse; Sigma-Aldrich) 24 h post infection. 96 h later T cell proliferation of the CFSE-labeled CD4^+^ T cells was analyzed by flow cytometry, as described above, after staining with pacific blue-conjugated anti-CD4 (eBiosciences), APC-Vα2 (eBiosciences), and 7-AAD.

For the *in vitro* stimulation, 1×10^4^ CFSE-labeled T cells were co-incubated with 1×10^3^ sorted splenic DCs per well in RPMI 1640 medium (Biochrom, Berlin, Germany) supplemented with 10% FBS (Sigma), 2 mM L-glutamine (Invitrogen), 100 U of penicillin/ml, 100 µg/ml of streptomycin (Biochrom), 50 µM β-mercaptoethanol (Sigma), 1% (vol/vol) nonessential amino acids (Biochrom), and 1 mM sodium pyruvate (Biochrom) in 96 V-bottom well plates in the presence of 100 µg/ml ovalbumin protein or 150 ng/ml OVA_323–339_ peptide. 72 h post incubation at 37°C cells were stained as described for the *in vivo* proliferation assay and acquired by flow cytometry on a Canto-II (BD Biosciences).

The division index was analyzed using FlowJo Software 7.5. It is defined as the average number of cell divisions that the responding cells underwent (excluding undivided cells).

### Measurement of antigen uptake and antigen processing by flow cytometry

For antigen uptake or antigen processing, 100 µg of ovalbumin-AlexaFluor647 and DQ-ovalbumin (both from Molecular Probes) were injected into the tail vain of mice infected for one or three days with Ye as described above. One h later the spleen of the mice was removed and processed for flow cytometry analyses as described above. The measurement of antigen uptake or processing was performed on a FACS Canto II flow cytometer (BD Biosciences) and analyzed using FlowJo Software 7.5 (Tree Star). The OVA processing was quantified as the frequency of OVA-Alexa647^+^OVADQ^+^cells/the frequency of OVA-Alexa647^+^ cells x 100.

### Immunofluorescence of cryosections

Tissues were embedded in the Tissue-Tek OCT compound (Sakura) and frozen at −80°C, and 5 µm cryostat sections were prepared. Tissue sections were fixed for 15 min at RT with 4% paraformaldehyde (TUNEL and CD11c staining) or for 10 min with ice cold acetone (CD11c staining), washed twice with PBS and the excess of biotin was blocked with a biotin blocking kit (Vector). Tissue sections were incubated overnight at 4°C with biotin-conjugated anti-CD11c antibody (HL3, 5 µg/ml in PBS-10% FBS), washed and then incubated with streptavidin-AlexaFluor594 (2.5 µg/ml in PBS-10% FBS) for 1 h at 4°C. Slides were further treated to visualize apoptotic cells using FITC-labeled TUNEL kit (Böhringer Mannheim) according to the manufacturer's instructions. Slides were mounted in Mowiol (Carl Roth). Labeled cells were visualized with a DMRE fluorescence microscope (Leica) or an Axiovert 200 M fluorescence microscope (Zeiss).

### Statistics

Data were analyzed using the Graph Pad Prism 4.0 software. Diagrams show mean values + SD, except for Fig. 6C where mean values + SEM are shown. Statistical analysis was performed using the unpaired two-tailed Student's *t* test. Differences were considered as statistically significant if p<0.05 (*), p<0.01 (**), or p<0.005 (***).

## Supporting Information

Figure S1 CCF4 stains living cells. Dot plots show untreated and staurosporine-treated (for three h at 37°C with 6 µM final concentration) splenocytes from C57BL/6 mice stained with 7-AAD and CCF4.
(0.08 MB TIF)

Figure S2 *In vitro* Ye infection of spleen cells does not result in changes of DC subpopulations. Dot plots show splenocytes from C57BL/6 mice untreated or infected *in vitro* for three days with Ye pYV^+^ (5×10^4^ bacteria/spleen) and stained for DC subpopulations as depicted in Fig. 5A.
(0.12 MB TIF)

Figure S3 Gating strategy for 7-AAD^+^ and caspases 3/7^+^ DCs. (A) Dot plots show gating of DC subpopulations including dead cells. (B) Histograms show analysis of 7-AAD^+^ DC subpopulations following the gating strategy shown in (A). The percentage above the marker was used for statistical analysis in Fig. 5C. Data are representative for 4 experiments with 5 mice per group. (C) Dot plots show the analysis of caspases 3/7^+^ DC subpopulations following the gating strategy shown in (A). The sum of the percentages in the lower right (caspases 3/7^+^ cells) and the upper right (caspases3/7^+^7-AAD^+^ cells) quadrants was used for statistical analysis in Fig. 5D. Data are representative for 2 independent experiments with 5 mice per group.
(0.46 MB TIF)
