# Supplementary figures and images for: Immune Evasion by Yersinia enterocolitica: Differential Targeting of Dendritic Cell Subpopulations In Vivo
Source: PLoS Pathog. 2010 Nov 24;6(11):e1001212. doi: 10.1371/journal.ppat.1001212 (PMC2991265; doi:10.1371/journal.ppat.1001212)

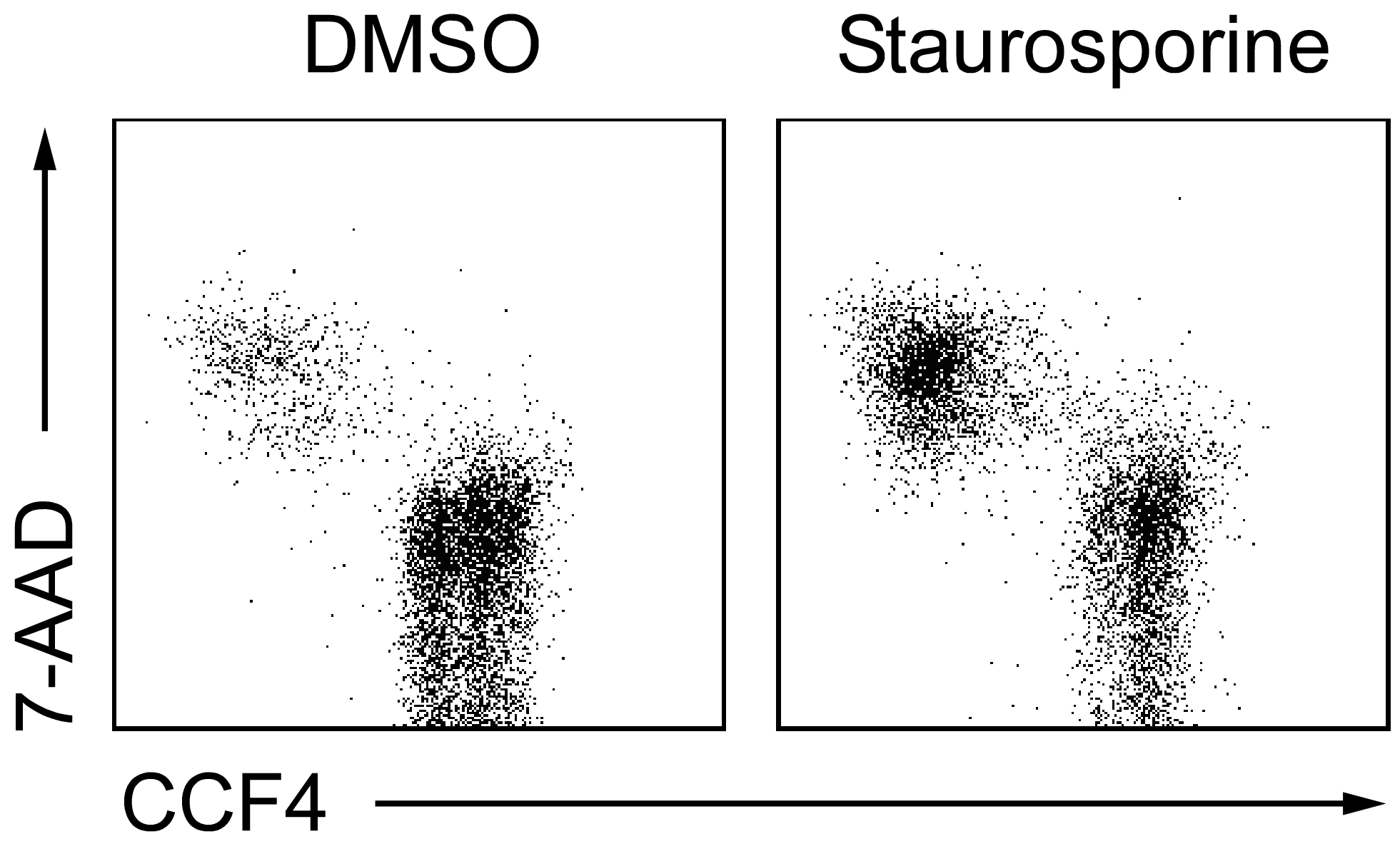

Supplement: Figure S1 — CCF4 stains living cells. Dot plots show untreated and staurosporine-treated (for three h at 37°C with 6 µM final concentration) splenocytes from C57BL/6 mice stained with 7-AAD and CCF4. (0.08 MB TIF) [file ppat.1001212.s001.tif]

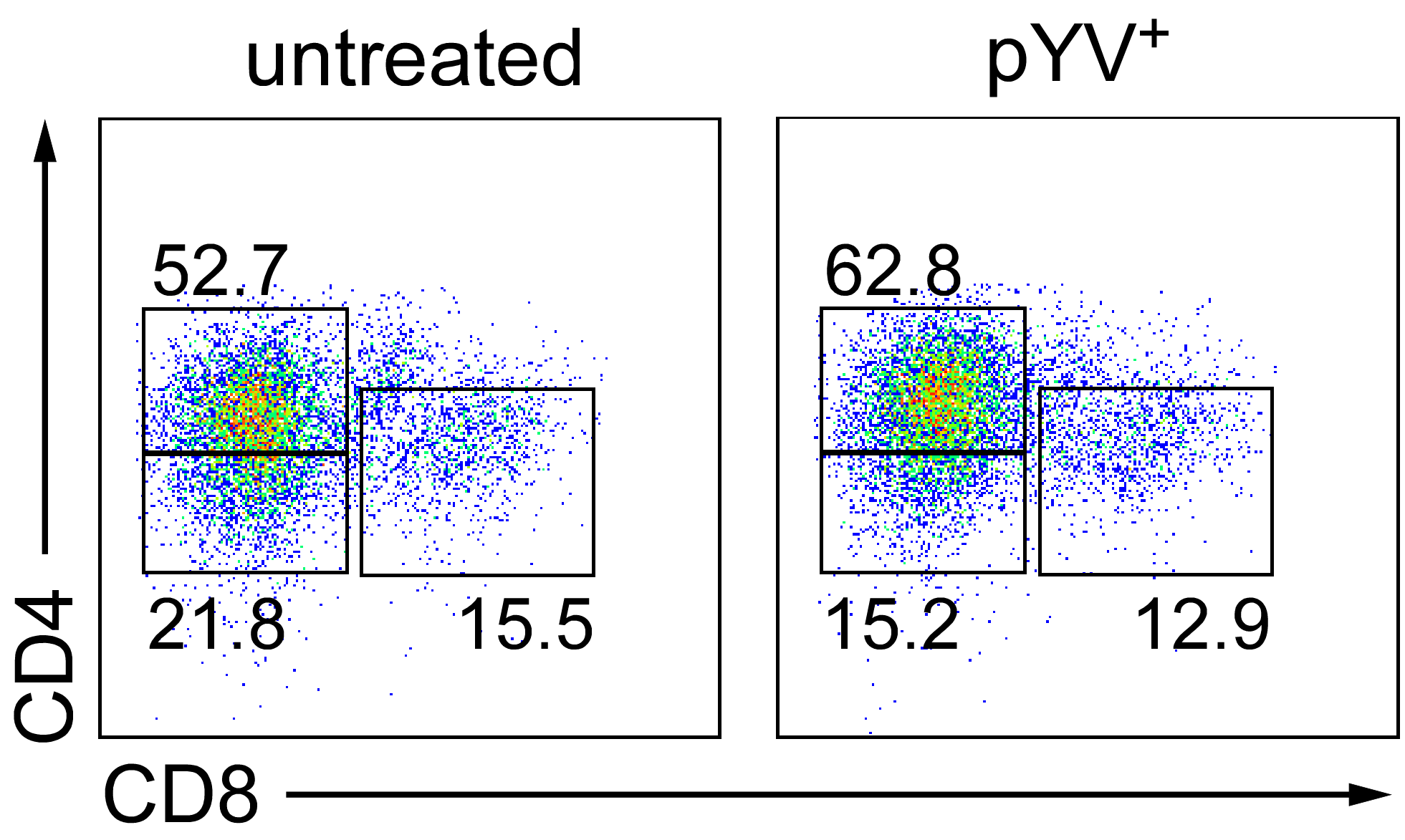

Supplement: Figure S2 — In vitro Ye infection of spleen cells does not result in changes of DC subpopulations. Dot plots show splenocytes from C57BL/6 mice untreated or infected in vitro for three days with Ye pYV+ (5×10^4^ bacteria/spleen) and stained for DC subpopulations as depicted in Fig. 5A. (0.12 MB TIF) [file ppat.1001212.s002.tif]

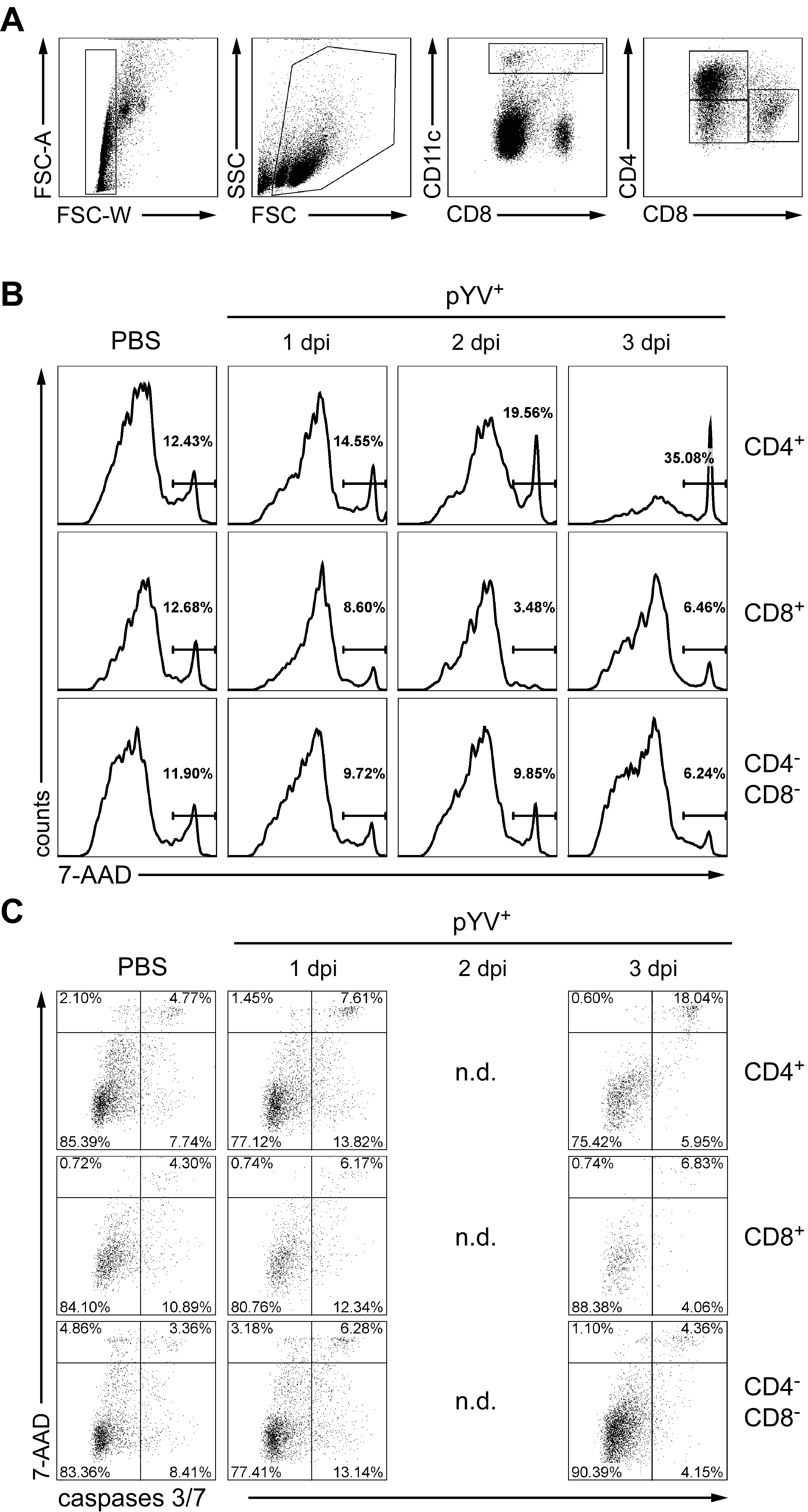

Supplement: Figure S3 — Gating strategy for 7-AAD+ and caspases 3/7+ DCs. (A) Dot plots show gating of DC subpopulations including dead cells. (B) Histograms show analysis of 7-AAD+ DC subpopulations following the gating strategy shown in (A). The percentage above the marker was used for statistical analysis in Fig. 5C. Data are representative for 4 experiments with 5 mice per group. (C) Dot plots show the analysis of caspases 3/7+ DC subpopulations following the gating strategy shown in (A). The sum of the percentages in the lower right (caspases 3/7+ cells) and the upper right (caspases3/7+7-AAD+ cells) quadrants was used for statistical analysis in Fig. 5D. Data are representative for 2 independent experiments with 5 mice per group. (0.46 MB TIF) [file ppat.1001212.s003.tif]
